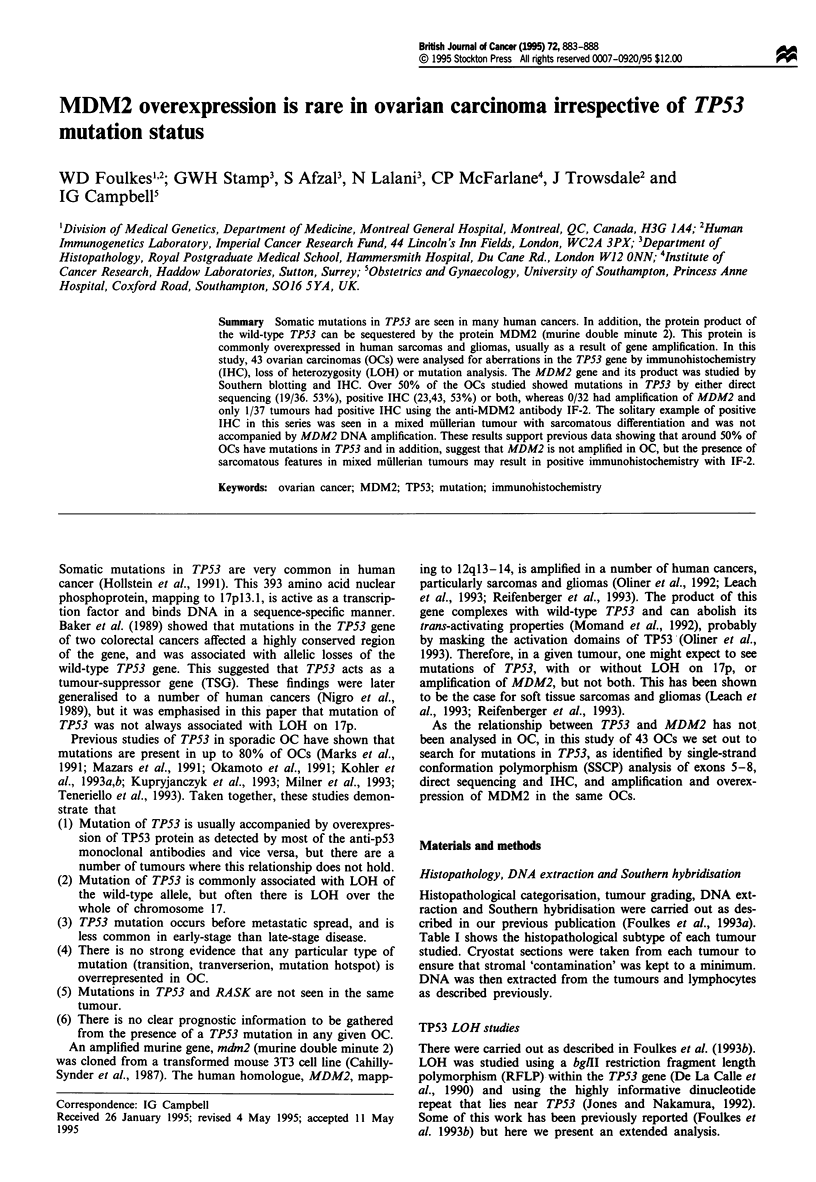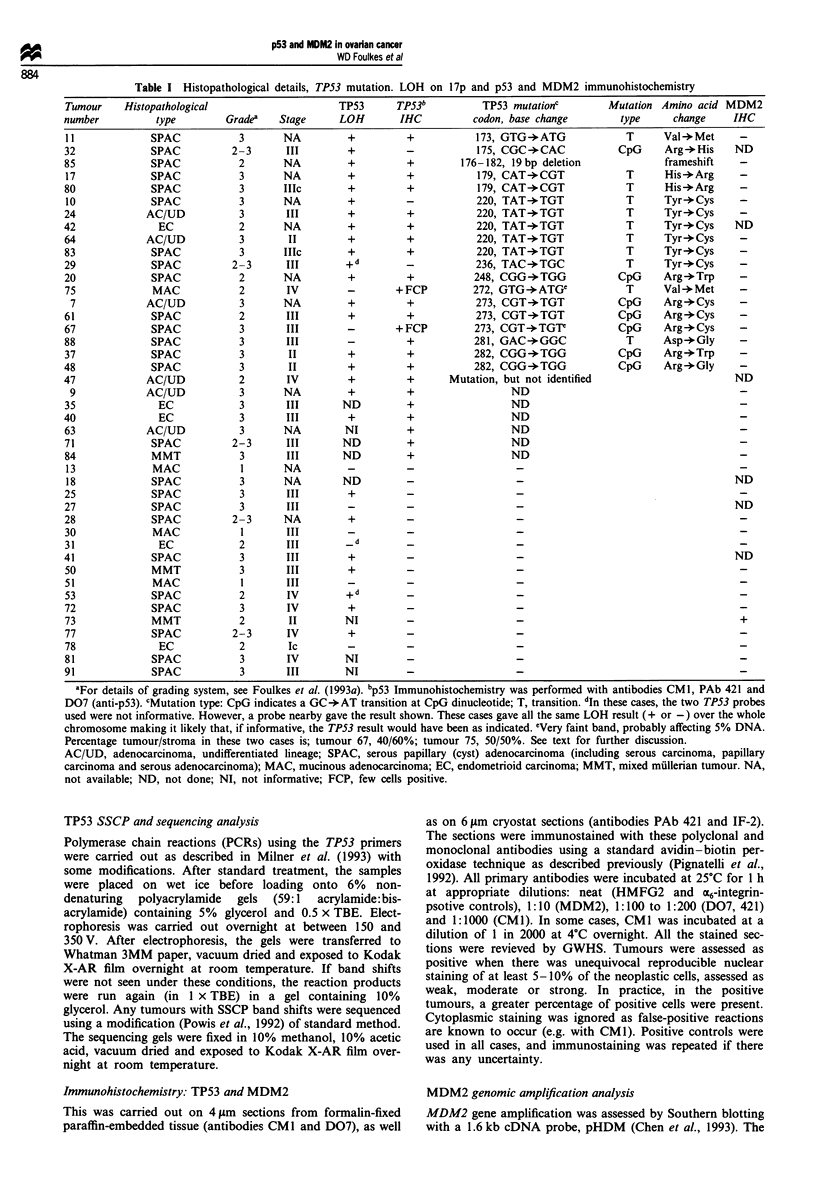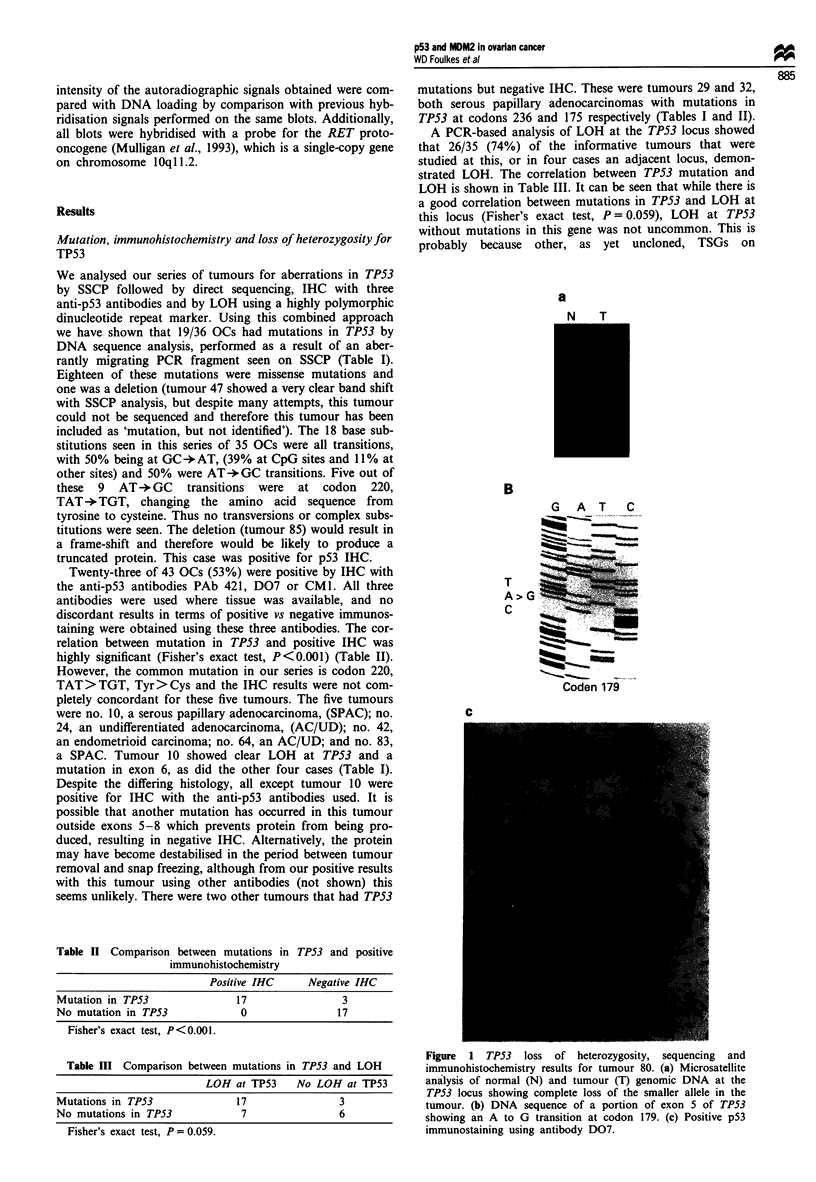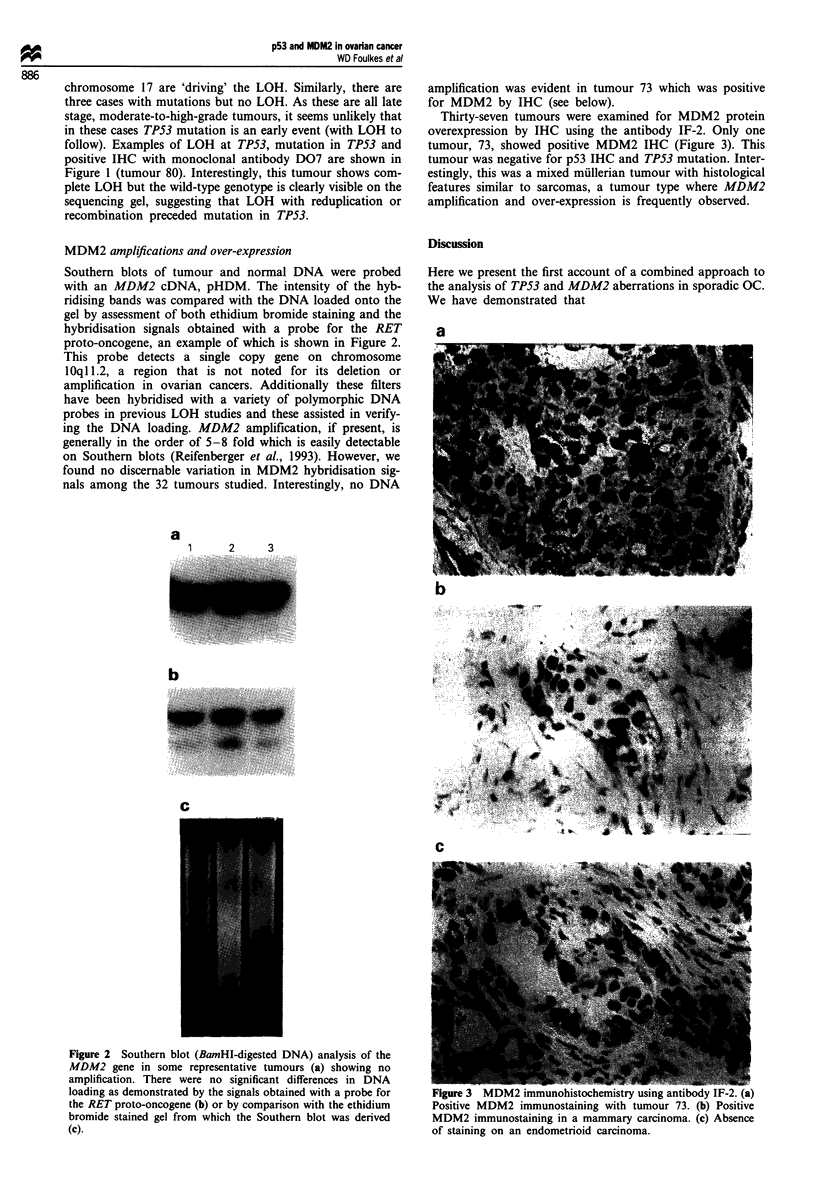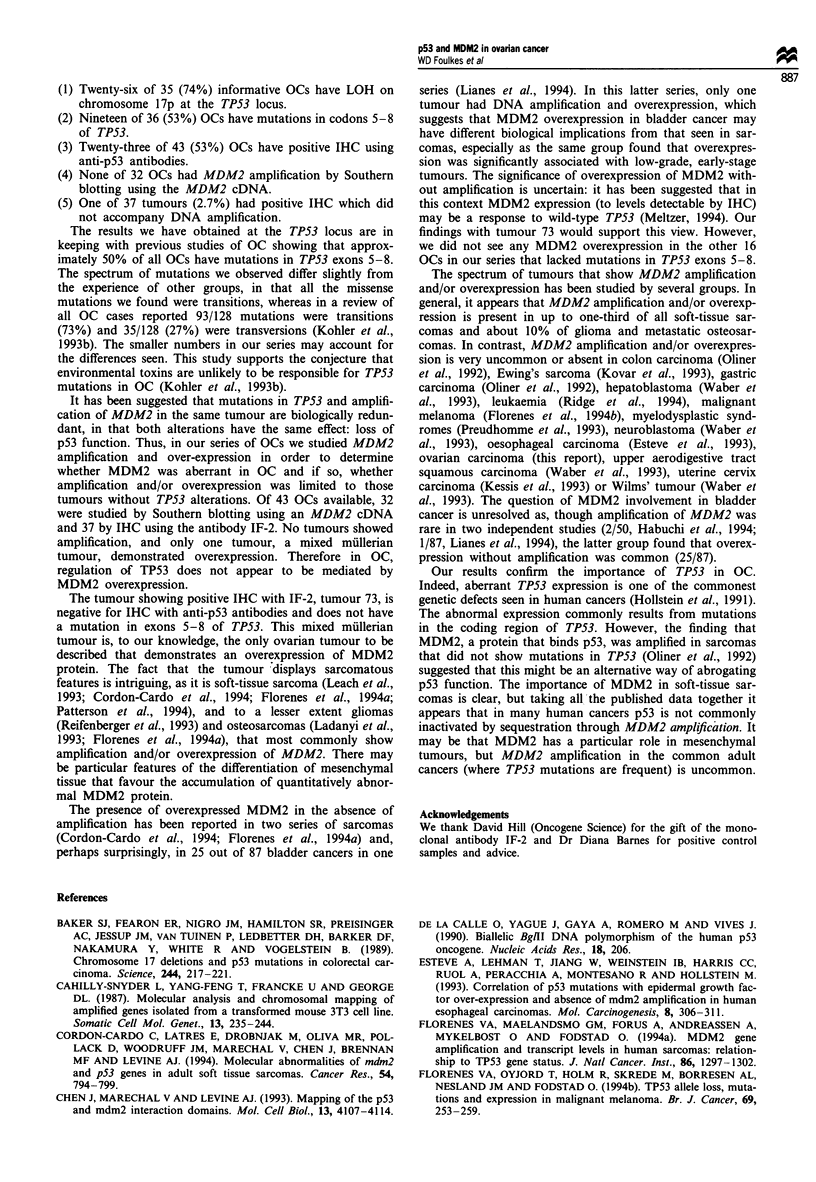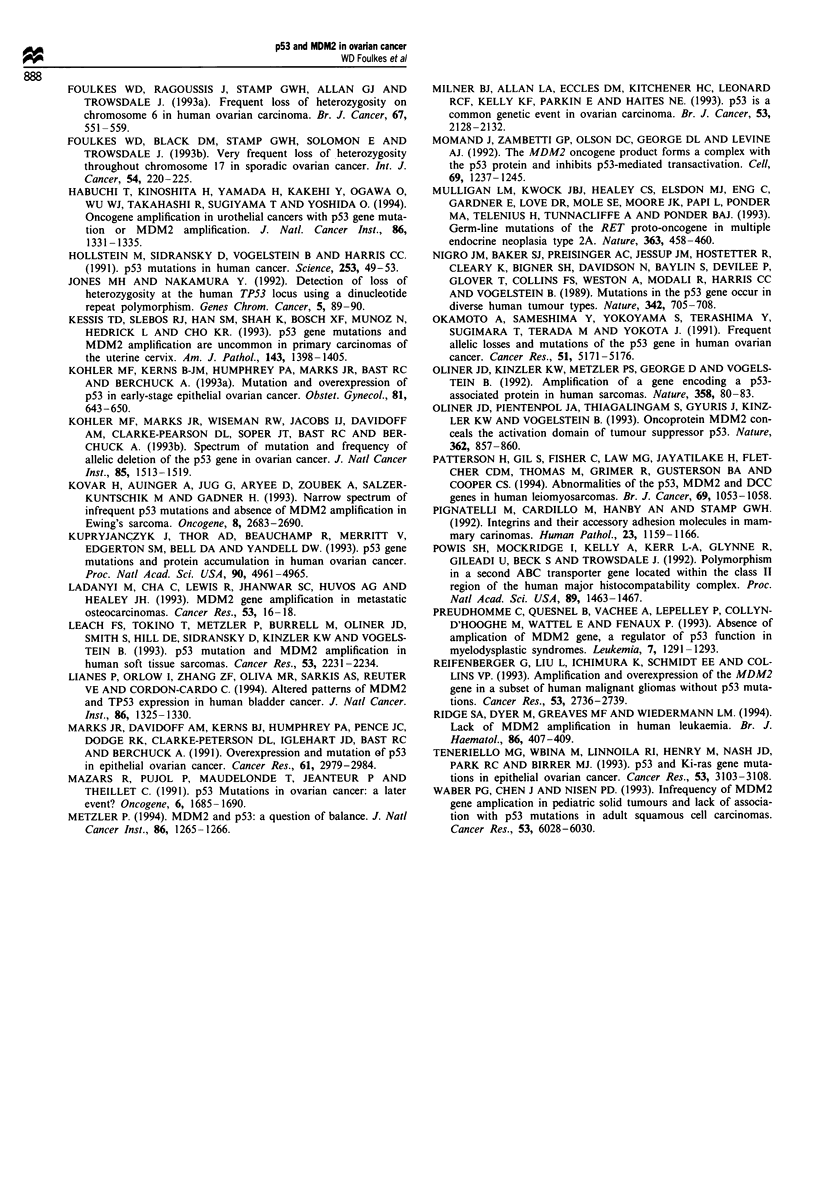# MDM2 overexpression is rare in ovarian carcinoma irrespective of TP53 mutation status.

**DOI:** 10.1038/bjc.1995.428

**Published:** 1995-10

**Authors:** W. D. Foulkes, G. W. Stamp, S. Afzal, N. Lalani, C. P. McFarlane, J. Trowsdale, I. G. Campbell

**Affiliations:** Department of Medicine, Montreal General Hospital, QC, Canada.

## Abstract

**Images:**


British Journal of Cancer (1995) 72, 883–888
© 1995 Stockton Press All rights reserved 0007-0920/95 $12.00

### MDM2 overexpression is rare in ovarian carcinoma irrespective of *TP53* mutation status

WD Foulkes[1,2]; GWH Stamp[3], S Afzal[3], N Lalani[3], CP McFarlane[4], J Trowsdale[2] and IG Campbell[5]

[1]*Division of Medical Genetics, Department of Medicine, Montreal General Hospital, Montreal, QC, Canada, H3G 1A4;* [2]*Human Immunogenetics Laboratory, Imperial Cancer Research Fund, 44 Lincoln's Inn Fields, London, WC2A 3PX;* [3]*Department of Histopathology, Royal Postgraduate Medical School, Hammersmith Hospital, Du Cane Rd., London W12 0NN;* [4]*Institute of Cancer Research, Haddow Laboratories, Sutton, Surrey;* [5]*Obstetrics and Gynaecology, University of Southampton, Princess Anne Hospital, Coxford Road, Southampton, SO16 5YA, UK.*

**Summary** Somatic mutations in *TP53* are seen in many human cancers. In addition, the protein product of the wild-type *TP53* can be sequestered by the protein MDM2 (murine double minute 2). This protein is commonly overexpressed in human sarcomas and gliomas, usually as a result of gene amplification. In this study, 43 ovarian carcinomas (OCs) were analysed for aberrations in the *TP53* gene by immunohistochemistry (IHC), loss of heterozygosity (LOH) or mutation analysis. The *MDM2* gene and its product was studied by Southern blotting and IHC. Over 50% of the OCs studied showed mutations in *TP53* by either direct sequencing (19/36. 53%), positive IHC (23,43, 53%) or both, whereas 0/32 had amplification of *MDM2* and only 1/37 tumours had positive IHC using the anti-MDM2 antibody IF-2. The solitary example of positive IHC in this series was seen in a mixed müllerian tumour with sarcomatous differentiation and was not accompanied by *MDM2* DNA amplification. These results support previous data showing that around 50% of OCs have mutations in *TP53* and in addition, suggest that *MDM2* is not amplified in OC, but the presence of sarcomatous features in mixed müllerian tumours may result in positive immunohistochemistry with IF-2.

**Keywords:** ovarian cancer; MDM2; TP53; mutation; immunohistochemistry

Somatic mutations in *TP53* are very common in human cancer (Hollstein *et al.*, 1991). This 393 amino acid nuclear phosphoprotein, mapping to 17p13.1, is active as a transcription factor and binds DNA in a sequence-specific manner. Baker *et al.* (1989) showed that mutations in the *TP53* gene of two colorectal cancers affected a highly conserved region of the gene, and was associated with allelic losses of the wild-type *TP53* gene. This suggested that *TP53* acts as a tumour-suppressor gene (TSG). These findings were later generalised to a number of human cancers (Nigro *et al.*, 1989), but it was emphasised in this paper that mutation of *TP53* was not always associated with LOH on 17p.

Previous studies of *TP53* in sporadic OC have shown that mutations are present in up to 80% of OCs (Marks *et al.*, 1991; Mazars *et al.*, 1991; Okamoto *et al.*, 1991; Kohler *et al.*, 1993*a,b*; Kupryjanczyk *et al.*, 1993; Milner *et al.*, 1993; Teneriello *et al.*, 1993). Taken together, these studies demonstrate that

(1) Mutation of *TP53* is usually accompanied by overexpression of TP53 protein as detected by most of the anti-p53 monoclonal antibodies and vice versa, but there are a number of tumours where this relationship does not hold.
(2) Mutation of *TP53* is commonly associated with LOH of the wild-type allele, but often there is LOH over the whole of chromosome 17.
(3) *TP53* mutation occurs before metastatic spread, and is less common in early-stage than late-stage disease.
(4) There is no strong evidence that any particular type of mutation (transition, tranverserion, mutation hotspot) is overrepresented in OC.
(5) Mutations in *TP53* and *RASK* are not seen in the same tumour.
(6) There is no clear prognostic information to be gathered from the presence of a *TP53* mutation in any given OC.

An amplified murine gene, *mdm2* (murine double minute 2) was cloned from a transformed mouse 3T3 cell line (Cahilly-Synder *et al.*, 1987). The human homologue, *MDM2*, mapping to 12q13–14, is amplified in a number of human cancers, particularly sarcomas and gliomas (Oliner *et al.*, 1992; Leach *et al.*, 1993; Reifenberger *et al.*, 1993). The product of this gene complexes with wild-type *TP53* and can abolish its *trans*-activating properties (Momand *et al.*, 1992), probably by masking the activation domains of TP53 (Oliner *et al.*, 1993). Therefore, in a given tumour, one might expect to see mutations of *TP53*, with or without LOH on 17p, or amplification of *MDM2*, but not both. This has been shown to be the case for soft tissue sarcomas and gliomas (Leach *et al.*, 1993; Reifenberger *et al.*, 1993).

As the relationship between *TP53* and *MDM2* has not been analysed in OC, in this study of 43 OCs we set out to search for mutations in *TP53*, as identified by single-strand conformation polymorphism (SSCP) analysis of exons 5–8, direct sequencing and IHC, and amplification and overexpression of MDM2 in the same OCs.

Correspondence: IG Campbell
Received 26 January 1995; revised 4 May 1995; accepted 11 May 1995

### Materials and methods

### Histopathology, DNA extraction and Southern hybridisation

Histopathological categorisation, tumour grading, DNA extraction and Southern hybridisation were carried out as described in our previous publication (Foulkes *et al.*, 1993*a*). Table I shows the histopathological subtype of each tumour studied. Cryostat sections were taken from each tumour to ensure that stromal 'contamination' was kept to a minimum. DNA was then extracted from the tumours and lymphocytes as described previously.

### TP53 *LOH studies*

There were carried out as described in Foulkes *et al.* (1993*b*). LOH was studied using a *bgl*II restriction fragment length polymorphism (RFLP) within the *TP53* gene (De La Calle *et al.*, 1990) and using the highly informative dinucleotide repeat that lies near *TP53* (Jones and Nakamura, 1992). Some of this work has been previously reported (Foulkes *et al.* 1993*b*) but here we present an extended analysis.

**Table I** Histopathological details, *TP53* mutation. LOH on 17p and p53 and MDM2 immunohistochemistry

| *Tumour number* | *Histopathological type* | *Grade*[a] | *Stage* | TP53 *LOH* | *TP53*[b] *IHC* | TP53 *mutation*[c] *codon, base change* | *Mutation type* | *Amino acid change* | MDM2 *IHC* |
|---|---|---|---|---|---|---|---|---|---|
| 11 | SPAC | 3 | NA | + | + | 173, GTG→ATG | T | Val→Met | – |
| 32 | SPAC | 2–3 | III | + | – | 175, CGC→CAC | CpG | Arg→His | ND |
| 85 | SPAC | 2 | NA | + | + | 176–182, 19 bp deletion | | frameshift | – |
| 17 | SPAC | 3 | NA | + | + | 179, CAT→CGT | T | His→Arg | – |
| 80 | SPAC | 3 | IIIc | + | + | 179, CAT→CGT | T | His→Arg | – |
| 10 | SPAC | 3 | NA | + | – | 220, TAT→TGT | T | Tyr→Cys | – |
| 24 | AC/UD | 3 | III | + | + | 220, TAT→TGT | T | Tyr→Cys | – |
| 42 | EC | 2 | NA | + | + | 220, TAT→TGT | T | Tyr→Cys | ND |
| 64 | AC/UD | 3 | II | + | + | 220, TAT→TGT | T | Tyr→Cys | – |
| 83 | SPAC | 3 | IIIc | + | + | 220, TAT→TGT | T | Tyr→Cys | – |
| 29 | SPAC | 2–3 | III | +[d] | – | 236, TAC→TGC | T | Tyr→Cys | – |
| 20 | SPAC | 2 | NA | + | + | 248, CGG→TGG | CpG | Arg→Trp | – |
| 75 | MAC | 2 | IV | – | +FCP | 272, GTG→ATG[e] | T | Val→Met | – |
| 7 | AC/UD | 3 | NA | + | + | 273, CGT→TGT | CpG | Arg→Cys | – |
| 61 | SPAC | 2 | III | + | + | 273, CGT→TGT | CpG | Arg→Cys | – |
| 67 | SPAC | 3 | III | – | +FCP | 273, CGT→TGT[e] | CpG | Arg→Cys | – |
| 88 | SPAC | 3 | III | – | + | 281, GAC→GGC | T | Asp→Gly | – |
| 37 | SPAC | 3 | II | + | + | 282, CGG→TGG | CpG | Arg→Trp | – |
| 48 | SPAC | 3 | II | + | + | 282, CGG→TGG | CpG | Arg→Gly | – |
| 47 | AC/UD | 2 | IV | + | + | Mutation, but not identified | | | ND |
| 9 | AC/UD | 3 | NA | + | + | ND | | | – |
| 35 | EC | 3 | III | ND | + | ND | | | – |
| 40 | EC | 3 | III | + | + | ND | | | – |
| 63 | AC/UD | 3 | NA | NI | + | ND | | | – |
| 71 | SPAC | 2–3 | III | ND | + | ND | | | – |
| 84 | MMT | 3 | III | ND | + | ND | | | – |
| 13 | MAC | 1 | NA | – | – | – | | | – |
| 18 | SPAC | 3 | NA | ND | – | – | | | ND |
| 25 | SPAC | 3 | III | + | – | – | | | – |
| 27 | SPAC | 3 | III | – | – | – | | | ND |
| 28 | SPAC | 2–3 | NA | + | – | – | | | – |
| 30 | MAC | 1 | III | – | – | – | | | – |
| 31 | EC | 2 | III | –[d] | – | – | | | – |
| 41 | SPAC | 3 | III | + | – | – | | | ND |
| 50 | MMT | 3 | III | + | – | – | | | – |
| 51 | MAC | 1 | III | – | – | – | | | – |
| 53 | SPAC | 2 | IV | +[d] | – | – | | | – |
| 72 | SPAC | 3 | IV | + | – | – | | | – |
| 73 | MMT | 2 | II | NI | – | – | | | + |
| 77 | SPAC | 2–3 | IV | + | – | – | | | – |
| 78 | EC | 2 | Ic | – | – | – | | | – |
| 81 | SPAC | 3 | IV | NI | – | – | | | – |
| 91 | SPAC | 3 | III | NI | – | – | | | – |

[a]For details of grading system, see Foulkes *et al.* (1993*a*). [b]p53 Immunohistochemistry was performed with antibodies CM1, PAb 421 and DO7 (anti-p53). [c]Mutation type: CpG indicates a GC→AT transition at CpG dinucleotide; T, transition. [d]In these cases, the two *TP53* probes used were not informative. However, a probe nearby gave the result shown. These cases gave all the same LOH result (+ or –) over the whole chromosome making it likely that, if informative, the *TP53* result would have been as indicated. [e]Very faint band, probably affecting 5% DNA. Percentage tumour/stroma in these two cases is; tumour 67, 40/60%; tumour 75, 50/50%. See text for further discussion.
AC/UD, adenocarcinoma, undifferentiated lineage; SPAC, serous papillary (cyst) adenocarcinoma (including serous carcinoma, papillary carcinoma and serous adenocarcinoma); MAC, mucinous adenocarcinoma; EC, endometrioid carcinoma; MMT, mixed müllerian tumour. NA, not available; ND, not done; NI, not informative; FCP, few cells positive.

### TP53 *SSCP and sequencing analysis*

Polymerase chain reactions (PCRs) using the *TP53* primers were carried out as described in Milner *et al.* (1993) with some modifications. After standard treatment, the samples were placed on wet ice before loading onto 6% non-denaturing polyacrylamide gels (59:1 acrylamide:bis-acrylamide) containing 5% glycerol and 0.5 × TBE. Electrophoresis was carried out overnight at between 150 and 350 V. After electrophoresis, the gels were transferred to Whatman 3MM paper, vacuum dried and exposed to Kodak X-AR film overnight at room temperature. If band shifts were not seen under these conditions, the reaction products were run again (in 1 × TBE) in a gel containing 10% glycerol. Any tumours with SSCP band shifts were sequenced using a modification (Powis *et al.*, 1992) of standard method. The sequencing gels were fixed in 10% methanol, 10% acetic acid, vacuum dried and exposed to Kodak X-AR film overnight at room temperature.

### *Immunohistochemistry:* TP53 *and* MDM2

This was carried out on 4 μm sections from formalin-fixed paraffin-embedded tissue (antibodies CM1 and DO7), as well as on 6 μm cryostat sections (antibodies PAb 421 and IF-2). The sections were immunostained with these polyclonal and monoclonal antibodies using a standard avidin–biotin peroxidase technique as described previously (Pignatelli *et al.*, 1992). All primary antibodies were incubated at 25°C for 1 h at appropriate dilutions: neat (HMFG2 and $\alpha_6$-integrin-psotive controls), 1:10 (MDM2), 1:100 to 1:200 (DO7, 421) and 1:1000 (CM1). In some cases, CM1 was incubated at a dilution of 1 in 2000 at 4°C overnight. All the stained sections were reviewed by GWHS. Tumours were assessed as positive when there was unequivocal reproducible nuclear staining of at least 5–10% of the neoplastic cells, assessed as weak, moderate or strong. In practice, in the positive tumours, a greater percentage of positive cells were present. Cytoplasmic staining was ignored as false-positive reactions are known to occur (e.g. with CM1). Positive controls were used in all cases, and immunostaining was repeated if there was any uncertainty.

### MDM2 *genomic amplification analysis*

*MDM2* gene amplification was assessed by Southern blotting with a 1.6 kb cDNA probe, pHDM (Chen *et al.*, 1993). The intensity of the autoradiographic signals obtained were compared with DNA loading by comparison with previous hybridisation signals performed on the same blots. Additionally, all blots were hybridised with a probe for the *RET* proto-oncogene (Mulligan *et al.*, 1993), which is a single-copy gene on chromosome 10q11.2.

### Results

### *Mutation, immunohistochemistry and loss of heterozygosity for* TP53

We analysed our series of tumours for aberrations in *TP53* by SSCP followed by direct sequencing, IHC with three anti-p53 antibodies and by LOH using a highly polymorphic dinucleotide repeat marker. Using this combined approach we have shown that 19/36 OCs had mutations in *TP53* by DNA sequence analysis, performed as a result of an aberrantly migrating PCR fragment seen on SSCP (Table I). Eighteen of these mutations were missense mutations and one was a deletion (tumour 47 showed a very clear band shift with SSCP analysis, but despite many attempts, this tumour could not be sequenced and therefore this tumour has been included as 'mutation, but not identified'). The 18 base substitutions seen in this series of 35 OCs were all transitions, with 50% being at GC→AT, (39% at CpG sites and 11% at other sites) and 50% were AT→GC transitions. Five out of these 9 AT→GC transitions were at codon 220, TAT→TGT, changing the amino acid sequence from tyrosine to cysteine. Thus no transversions or complex substitutions were seen. The deletion (tumour 85) would result in a frame-shift and therefore would be likely to produce a truncated protein. This case was positive for p53 IHC.

Twenty-three of 43 OCs (53%) were positive by IHC with the anti-p53 antibodies PAb 421, DO7 or CM1. All three antibodies were used where tissue was available, and no discordant results in terms of positive *vs* negative immunostaining were obtained using these three antibodies. The correlation between mutation in *TP53* and positive IHC was highly significant (Fisher's exact test, $P<0.001$) (Table II). However, the common mutation in our series is codon 220, TAT>TGT, Tyr>Cys and the IHC results were not completely concordant for these five tumours. The five tumours were no. 10, a serous papillary adenocarcinoma, (SPAC); no. 24, an undifferentiated adenocarcinoma, (AC/UD); no. 42, an endometrioid carcinoma; no. 64, an AC/UD; and no. 83, a SPAC. Tumour 10 showed clear LOH at *TP53* and a mutation in exon 6, as did the other four cases (Table I). Despite the differing histology, all except tumour 10 were positive for IHC with the anti-p53 antibodies used. It is possible that another mutation has occurred in this tumour outside exons 5–8 which prevents protein from being produced, resulting in negative IHC. Alternatively, the protein may have become destabilised in the period between tumour removal and snap freezing, although from our positive results with this tumour using other antibodies (not shown) this seems unlikely. There were two other tumours that had *TP53* mutations but negative IHC. These were tumours 29 and 32, both serous papillary adenocarcinomas with mutations in *TP53* at codons 236 and 175 respectively (Tables I and II).

A PCR-based analysis of LOH at the *TP53* locus showed that 26/35 (74%) of the informative tumours that were studied at this, or in four cases an adjacent locus, demonstrated LOH. The correlation between *TP53* mutation and LOH is shown in Table III. It can be seen that while there is a good correlation between mutations in *TP53* and LOH at this locus (Fisher's exact test, $P=0.059$), LOH at *TP53* without mutations in this gene was not uncommon. This is probably because other, as yet uncloned, TSGs on

**Table II** Comparison between mutations in *TP53* and positive immunohistochemistry

| | *Positive IHC* | *Negative IHC* |
|---|---|---|
| Mutation in *TP53* | 17 | 3 |
| No mutation in *TP53* | 0 | 17 |

Fisher's exact test, $P<0.001$.

**Table III** Comparison between mutations in *TP53* and LOH

| | *LOH at* TP53 | *No LOH at* TP53 |
|---|---|---|
| Mutations in *TP53* | 17 | 3 |
| No mutations in *TP53* | 7 | 6 |

Fisher's exact test, $P=0.059$.

**Figure 1** *TP53* loss of heterozygosity, sequencing and immunohistochemistry results for tumour 80. (**a**) Microsatellite analysis of normal (N) and tumour (T) genomic DNA at the *TP53* locus showing complete loss of the smaller allele in the tumour. (**b**) DNA sequence of a portion of exon 5 of *TP53* showing an A to G transition at codon 179. (**c**) Positive p53 immunostaining using antibody DO7.

chromosome 17 are 'driving' the LOH. Similarly, there are three cases with mutations but no LOH. As these are all late stage, moderate-to-high-grade tumours, it seems unlikely that in these cases *TP53* mutation is an early event (with LOH to follow). Examples of LOH at *TP53*, mutation in *TP53* and positive IHC with monoclonal antibody DO7 are shown in Figure 1 (tumour 80). Interestingly, this tumour shows complete LOH but the wild-type genotype is clearly visible on the sequencing gel, suggesting that LOH with reduplication or recombination preceded mutation in *TP53*.

### MDM2 *amplifications and over-expression*

Southern blots of tumour and normal DNA were probed with an *MDM2* cDNA, pHDM. The intensity of the hybridising bands was compared with the DNA loaded onto the gel by assessment of both ethidium bromide staining and the hybridisation signals obtained with a probe for the *RET* proto-oncogene, an example of which is shown in Figure 2. This probe detects a single copy gene on chromosome 10q11.2, a region that is not noted for its deletion or amplification in ovarian cancers. Additionally these filters have been hybridised with a variety of polymorphic DNA probes in previous LOH studies and these assisted in verifying the DNA loading. *MDM2* amplification, if present, is generally in the order of 5–8 fold which is easily detectable on Southern blots (Reifenberger *et al*., 1993). However, we found no discernable variation in MDM2 hybridisation signals among the 32 tumours studied. Interestingly, no DNA amplification was evident in tumour 73 which was positive for MDM2 by IHC (see below).

Thirty-seven tumours were examined for MDM2 protein overexpression by IHC using the antibody IF-2. Only one tumour, 73, showed positive MDM2 IHC (Figure 3). This tumour was negative for p53 IHC and *TP53* mutation. Interestingly, this was a mixed müllerian tumour with histological features similar to sarcomas, a tumour type where *MDM2* amplification and over-expression is frequently observed.

### Discussion

Here we present the first account of a combined approach to the analysis of *TP53* and *MDM2* aberrations in sporadic OC. We have demonstrated that

**Figure 2** Southern blot (*Bam*HI-digested DNA) analysis of the *MDM2* gene in some representative tumours (**a**) showing no amplification. There were no significant differences in DNA loading as demonstrated by the signals obtained with a probe for the *RET* proto-oncogene (**b**) or by comparison with the ethidium bromide stained gel from which the Southern blot was derived (**c**).

**Figure 3** MDM2 immunohistochemistry using antibody IF-2. (**a**) Positive MDM2 immunostaining with tumour 73. (**b**) Positive MDM2 immunostaining in a mammary carcinoma. (**c**) Absence of staining on an endometrioid carcinoma.

(1) Twenty-six of 35 (74%) informative OCs have LOH on chromosome 17p at the *TP53* locus.
(2) Nineteen of 36 (53%) OCs have mutations in codons 5–8 of *TP53*.
(3) Twenty-three of 43 (53%) OCs have positive IHC using anti-p53 antibodies.
(4) None of 32 OCs had *MDM2* amplification by Southern blotting using the *MDM2* cDNA.
(5) One of 37 tumours (2.7%) had positive IHC which did not accompany DNA amplification.

The results we have obtained at the *TP53* locus are in keeping with previous studies of OC showing that approximately 50% of all OCs have mutations in *TP53* exons 5–8. The spectrum of mutations we observed differ slightly from the experience of other groups, in that all the missense mutations we found were transitions, whereas in a review of all OC cases reported 93/128 mutations were transitions (73%) and 35/128 (27%) were transversions (Kohler *et al.*, 1993b). The smaller numbers in our series may account for the differences seen. This study supports the conjecture that environmental toxins are unlikely to be responsible for *TP53* mutations in OC (Kohler *et al.*, 1993b).

It has been suggested that mutations in *TP53* and amplification of *MDM2* in the same tumour are biologically redundant, in that both alterations have the same effect: loss of p53 function. Thus, in our series of OCs we studied *MDM2* amplification and over-expression in order to determine whether MDM2 was aberrant in OC and if so, whether amplification and/or overexpression was limited to those tumours without *TP53* alterations. Of 43 OCs available, 32 were studied by Southern blotting using an *MDM2* cDNA and 37 by IHC using the antibody IF-2. No tumours showed amplification, and only one tumour, a mixed müllerian tumour, demonstrated overexpression. Therefore in OC, regulation of TP53 does not appear to be mediated by MDM2 overexpression.

The tumour showing positive IHC with IF-2, tumour 73, is negative for IHC with anti-p53 antibodies and does not have a mutation in exons 5–8 of *TP53*. This mixed müllerian tumour is, to our knowledge, the only ovarian tumour to be described that demonstrates an overexpression of MDM2 protein. The fact that the tumour displays sarcomatous features is intriguing, as it is soft-tissue sarcoma (Leach *et al.*, 1993; Cordon-Cardo *et al.*, 1994; Florenes *et al.*, 1994*a*; Patterson *et al.*, 1994), and to a lesser extent gliomas (Reifenberger *et al.*, 1993) and osteosarcomas (Ladanyi *et al.*, 1993; Florenes *et al.*, 1994*a*), that most commonly show amplification and/or overexpression of *MDM2*. There may be particular features of the differentiation of mesenchymal tissue that favour the accumulation of quantitatively abnormal MDM2 protein.

The presence of overexpressed MDM2 in the absence of amplification has been reported in two series of sarcomas (Cordon-Cardo *et al.*, 1994; Florenes *et al.*, 1994*a*) and, perhaps surprisingly, in 25 out of 87 bladder cancers in one series (Lianes *et al.*, 1994). In this latter series, only one tumour had DNA amplification and overexpression, which suggests that MDM2 overexpression in bladder cancer may have different biological implications from that seen in sarcomas, especially as the same group found that overexpression was significantly associated with low-grade, early-stage tumours. The significance of overexpression of MDM2 without amplification is uncertain: it has been suggested that in this context MDM2 expression (to levels detectable by IHC) may be a response to wild-type *TP53* (Meltzer, 1994). Our findings with tumour 73 would support this view. However, we did not see any MDM2 overexpression in the other 16 OCs in our series that lacked mutations in *TP53* exons 5–8.

The spectrum of tumours that show *MDM2* amplification and/or overexpression has been studied by several groups. In general, it appears that *MDM2* amplification and/or overexpression is present in up to one-third of all soft-tissue sarcomas and about 10% of glioma and metastatic osteosarcomas. In contrast, *MDM2* amplification and/or overexpression is very uncommon or absent in colon carcinoma (Oliner *et al.*, 1992), Ewing's sarcoma (Kovar *et al.*, 1993), gastric carcinoma (Oliner *et al.*, 1992), hepatoblastoma (Waber *et al.*, 1993), leukaemia (Ridge *et al.*, 1994), malignant melanoma (Florenes *et al.*, 1994*b*), myelodysplastic syndromes (Preudhomme *et al.*, 1993), neuroblastoma (Waber *et al.*, 1993), oesophageal carcinoma (Esteve *et al.*, 1993), ovarian carcinoma (this report), upper aerodigestive tract squamous carcinoma (Waber *et al.*, 1993), uterine cervix carcinoma (Kessis *et al.*, 1993) or Wilms' tumour (Waber *et al.*, 1993). The question of MDM2 involvement in bladder cancer is unresolved as, though amplification of *MDM2* was rare in two independent studies (2/50, Habuchi *et al.*, 1994; 1/87, Lianes *et al.*, 1994), the latter group found that overexpression without amplification was common (25/87).

Our results confirm the importance of *TP53* in OC. Indeed, aberrant *TP53* expression is one of the commonest genetic defects seen in human cancers (Hollstein *et al.*, 1991). The abnormal expression commonly results from mutations in the coding region of *TP53*. However, the finding that MDM2, a protein that binds p53, was amplified in sarcomas that did not show mutations in *TP53* (Oliner *et al.*, 1992) suggested that this might be an alternative way of abrogating p53 function. The importance of MDM2 in soft-tissue sarcomas is clear, but taking all the published data together it appears that in many human cancers p53 is not commonly inactivated by sequestration through *MDM2 amplification*. It may be that MDM2 has a particular role in mesenchymal tumours, but *MDM2* amplification in the common adult cancers (where *TP53* mutations are frequent) is uncommon.

**Acknowledgements**
We thank David Hill (Oncogene Science) for the gift of the monoclonal antibody IF-2 and Dr Diana Barnes for positive control samples and advice.

### References

BAKER SJ, FEARON ER, NIGRO JM, HAMILTON SR, PREISINGER AC, JESSUP JM, VAN TUINEN P, LEDBETTER DH, BARKER DF, NAKAMURA Y, WHITE R AND VOGELSTEIN B. (1989). Chromosome 17 deletions and p53 mutations in colorectal carcinoma. *Science*, **244**, 217–221.

CAHILLY-SNYDER L, YANG-FENG T, FRANCKE U AND GEORGE DL. (1987). Molecular analysis and chromosomal mapping of amplified genes isolated from a transformed mouse 3T3 cell line. *Somatic Cell Mol. Genet.*, **13**, 235–244.

CORDON-CARDO C, LATRES E, DROBNJAK M, OLIVA MR, POLLACK D, WOODRUFF JM, MARECHAL V, CHEN J, BRENNAN MF AND LEVINE AJ. (1994). Molecular abnormalities of *mdm2* and *p53* genes in adult soft tissue sarcomas. *Cancer Res.*, **54**, 794–799.

CHEN J, MARECHAL V AND LEVINE AJ. (1993). Mapping of the p53 and mdm2 interaction domains. *Mol. Cell Biol.*, **13**, 4107–4114.

DE LA CALLE O, YAGUE J, GAYA A, ROMERO M AND VIVES J. (1990). Biallelic *Bgl*II DNA polymorphism of the human p53 oncogene. *Nucleic Acids Res.*, **18**, 206.

ESTEVE A, LEHMAN T, JIANG W, WEINSTEIN IB, HARRIS CC, RUOL A, PERACCHIA A, MONTESANO R AND HOLLSTEIN M. (1993). Correlation of p53 mutations with epidermal growth factor over-expression and absence of mdm2 amplification in human esophageal carcinomas. *Mol. Carcinogenesis*, **8**, 306–311.

FLORENES VA, MAELANDSMO GM, FORUS A, ANDREASSEN A, MYKELBOST O AND FODSTAD O. (1994a). MDM2 gene amplification and transcript levels in human sarcomas: relationship to TP53 gene status. *J. Natl Cancer. Inst.*, **86**, 1297–1302.

FLORENES VA, OYJORD T, HOLM R, SKREDE M, BORRESEN AL, NESLAND JM AND FODSTAD O. (1994b). TP53 allele loss, mutations and expression in malignant melanoma. *Br. J. Cancer*, **69**, 253–259.

FOULKES WD, RAGOUSSIS J, STAMP GWH, ALLAN GJ AND TROWSDALE J. (1993a). Frequent loss of heterozygosity on chromosome 6 in human ovarian carcinoma. *Br. J. Cancer*, **67**, 551-559.

FOULKES WD, BLACK DM, STAMP GWH, SOLOMON E AND TROWSDALE J. (1993b). Very frequent loss of heterozygosity throughout chromosome 17 in sporadic ovarian cancer. *Int. J. Cancer*, **54**, 220-225.

HABUCHI T, KINOSHITA H, YAMADA H, KAKEHI Y, OGAWA O, WU WJ, TAKAHASHI R, SUGIYAMA T AND YOSHIDA O. (1994). Oncogene amplification in urothelial cancers with p53 gene mutation or MDM2 amplification. *J. Natl. Cancer Inst.*, **86**, 1331-1335.

HOLLSTEIN M, SIDRANSKY D, VOGELSTEIN B AND HARRIS CC. (1991). p53 mutations in human cancer. *Science*, **253**, 49-53.

JONES MH AND NAKAMURA Y. (1992). Detection of loss of heterozygosity at the human *TP53* locus using a dinucleotide repeat polymorphism. *Genes Chrom. Cancer*, **5**, 89-90.

KESSIS TD, SLEBOS RJ, HAN SM, SHAH K, BOSCH XF, MUNOZ N, HEDRICK L AND CHO KR. (1993). p53 gene mutations and MDM2 amplification are uncommon in primary carcinomas of the uterine cervix. *Am. J. Pathol.*, **143**, 1398-1405.

KOHLER MF, KERNS B-JM, HUMPHREY PA, MARKS JR, BAST RC AND BERCHUCK A. (1993a). Mutation and overexpression of p53 in early-stage epithelial ovarian cancer. *Obstet. Gynecol.*, **81**, 643-650.

KOHLER MF, MARKS JR, WISEMAN RW, JACOBS IJ, DAVIDOFF AM, CLARKE-PEARSON DL, SOPER JT, BAST RC AND BERCHUCK A. (1993b). Spectrum of mutation and frequency of allelic deletion of the p53 gene in ovarian cancer. *J. Natl Cancer Inst.*, **85**, 1513-1519.

KOVAR H, AUINGER A, JUG G, ARYEE D, ZOUBEK A, SALZER-KUNTSCHIK M AND GADNER H. (1993). Narrow spectrum of infrequent p53 mutations and absence of MDM2 amplification in Ewing's sarcoma. *Oncogene*, **8**, 2683-2690.

KUPRYJANCZYK J, THOR AD, BEAUCHAMP R, MERRITT V, EDGERTON SM, BELL DA AND YANDELL DW. (1993). p53 gene mutations and protein accumulation in human ovarian cancer. *Proc. Natl Acad. Sci. USA*, **90**, 4961-4965.

LADANYI M, CHA C, LEWIS R, JHANWAR SC, HUVOS AG AND HEALEY JH. (1993). MDM2 gene amplification in metastatic osteocarcinomas. *Cancer Res.*, **53**, 16-18.

LEACH FS, TOKINO T, METZLER P, BURRELL M, OLINER JD, SMITH S, HILL DE, SIDRANSKY D, KINZLER KW AND VOGELSTEIN B. (1993). p53 mutation and MDM2 amplification in human soft tissue sarcomas. *Cancer Res.*, **53**, 2231-2234.

LIANES P, ORLOW I, ZHANG ZF, OLIVA MR, SARKIS AS, REUTER VE AND CORDON-CARDO C. (1994). Altered patterns of MDM2 and TP53 expression in human bladder cancer. *J. Natl Cancer. Inst.*, **86**, 1325-1330.

MARKS JR, DAVIDOFF AM, KERNS BJ, HUMPHREY PA, PENCE JC, DODGE RK, CLARKE-PETERSON DL, IGLEHART JD, BAST RC AND BERCHUCK A. (1991). Overexpression and mutation of p53 in epithelial ovarian cancer. *Cancer Res.*, **61**, 2979-2984.

MAZARS R, PUJOL P, MAUDELONDE T, JEANTEUR P AND THEILLET C. (1991). p53 Mutations in ovarian cancer: a later event? *Oncogene*, **6**, 1685-1690.

METZLER P. (1994). MDM2 and p53: a question of balance. *J. Natl Cancer Inst.*, **86**, 1265-1266.

MILNER BJ, ALLAN LA, ECCLES DM, KITCHENER HC, LEONARD RCF, KELLY KF, PARKIN E AND HAITES NE. (1993). p53 is a common genetic event in ovarian carcinoma. *Br. J. Cancer*, **53**, 2128-2132.

MOMAND J, ZAMBETTI GP, OLSON DC, GEORGE DL AND LEVINE AJ. (1992). The *MDM2* oncogene product forms a complex with the p53 protein and inhibits p53-mediated transactivation. *Cell*, **69**, 1237-1245.

MULLIGAN LM, KWOCK JBJ, HEALEY CS, ELSDON MJ, ENG C, GARDNER E, LOVE DR, MOLE SE, MOORE JK, PAPI L, PONDER MA, TELENIUS H, TUNNACLIFFE A AND PONDER BAJ. (1993). Germ-line mutations of the *RET* proto-oncogene in multiple endocrine neoplasia type 2A. *Nature*, **363**, 458-460.

NIGRO JM, BAKER SJ, PREISINGER AC, JESSUP JM, HOSTETTER R, CLEARY K, BIGNER SH, DAVIDSON N, BAYLIN S, DEVILEE P, GLOVER T, COLLINS FS, WESTON A, MODALI R, HARRIS CC AND VOGELSTEIN B. (1989). Mutations in the p53 gene occur in diverse human tumour types. *Nature*, **342**, 705-708.

OKAMOTO A, SAMESHIMA Y, YOKOYAMA S, TERASHIMA Y, SUGIMARA T, TERADA M AND YOKOTA J. (1991). Frequent allelic losses and mutations of the p53 gene in human ovarian cancer. *Cancer Res.*, **51**, 5171-5176.

OLINER JD, KINZLER KW, METZLER PS, GEORGE D AND VOGELSTEIN B. (1992). Amplification of a gene encoding a p53-associated protein in human sarcomas. *Nature*, **358**, 80-83.

OLINER JD, PIENTENPOL JA, THIAGALINGAM S, GYURIS J, KINZLER KW AND VOGELSTEIN B. (1993). Oncoprotein MDM2 conceals the activation domain of tumour suppressor p53. *Nature*, **362**, 857-860.

PATTERSON H, GIL S, FISHER C, LAW MG, JAYATILAKE H, FLETCHER CDM, THOMAS M, GRIMER R, GUSTERSON BA AND COOPER CS. (1994). Abnormalities of the p53, MDM2 and DCC genes in human leiomyosarcomas. *Br. J. Cancer*, **69**, 1053-1058.

PIGNATELLI M, CARDILLO M, HANBY AN AND STAMP GWH. (1992). Integrins and their accessory adhesion molecules in mammary carinomas. *Human Pathol.*, **23**, 1159-1166.

POWIS SH, MOCKRIDGE I, KELLY A, KERR L-A, GLYNNE R, GILEADI U, BECK S AND TROWSDALE J. (1992). Polymorphism in a second ABC transporter gene located within the class II region of the human major histocompatability complex. *Proc. Natl Acad. Sci. USA*, **89**, 1463-1467.

PREUDHOMME C, QUESNEL B, VACHEE A, LEPELLEY P, COLLYN-D'HOOGHE M, WATTEL E AND FENAUX P. (1993). Absence of amplication of MDM2 gene, a regulator of p53 function in myelodysplastic syndromes. *Leukemia*, **7**, 1291-1293.

REIFENBERGER G, LIU L, ICHIMURA K, SCHMIDT EE AND COLLINS VP. (1993). Amplification and overexpression of the *MDM2* gene in a subset of human malignant gliomas without p53 mutations. *Cancer Res.*, **53**, 2736-2739.

RIDGE SA, DYER M, GREAVES MF AND WIEDERMANN LM. (1994). Lack of MDM2 amplification in human leukaemia. *Br. J. Haematol.*, **86**, 407-409.

TENERIELLO MG, WBINA M, LINNOILA RI, HENRY M, NASH JD, PARK RC AND BIRRER MJ. (1993). p53 and Ki-ras gene mutations in epithelial ovarian cancer. *Cancer Res.*, **53**, 3103-3108.

WABER PG, CHEN J AND NISEN PD. (1993). Infrequency of MDM2 gene amplication in pediatric solid tumours and lack of association with p53 mutations in adult squamous cell carcinomas. *Cancer Res.*, **53**, 6028-6030.